# Extracellular Vesicle-Derived DNA vs. CfDNA as a Biomarker for the Detection of Colon Cancer

**DOI:** 10.3390/genes12081171

**Published:** 2021-07-29

**Authors:** Kavita Thakur, Manu Smriti Singh, Sara Feldstein-Davydova, Victoria Hannes, Dov Hershkovitz, Shlomo Tsuriel

**Affiliations:** 1 Department of Pathology, Tel Aviv Sourasky Medical Center, Tel Aviv 62431, Israel; drkavitathakur20@gmail.com (K.T.); sarafe@tlvmc.gov.il (S.F.-D.); victoriaha@tlvmc.gov.il (V.H.); dovh@tlvmc.gov.il (D.H.); 2 Sackler Faculty of Medicine, Tel Aviv University, Tel Aviv 69978, Israel; 3 Laboratory of Precision Nano Medicine, Tel Aviv University, Tel Aviv 69978, Israel; Manu.Singh@bennett.edu.in; 4 School of Molecular Cell Biology & Biotechnology, George S. Wise Faculty of Life Sciences, Tel Aviv University, Tel Aviv 69978, Israel; 5 Department of Materials Sciences & Engineering, Iby and Aladar Fleischman Faculty of Engineering, Tel Aviv University, Tel Aviv 69978, Israel; 6 Center for Nanoscience and Nanotechnology, and Tel Aviv University, Tel Aviv 69978, Israel; 7 Cancer Biology Research Center, Tel Aviv University, Tel Aviv 69978, Israel; 8 Department of Biotechnology, School of Engineering and Applied Sciences, Bennett University, Tech Zone 2, Greater Noida 201310, India

**Keywords:** liquid biopsy, colon cancer, cfDNA, ctDNA, EV-DNA

## Abstract

Liquid biopsy has emerged as a promising non-invasive way to diagnose tumor and monitor its progression. Different types of liquid biopsies have different advantages and limitations. In the present research, we compared the use of two types of liquid biopsy, extracellular vesicle-derived DNA (EV-DNA) and cell-free DNA (cfDNA) for identifying tumor mutations in patients with colon carcinoma. Method: DNA was extracted from the tumor tissue of 33 patients diagnosed with colon carcinoma. Targeted NGS panel, based on the hotspots panel, was used to identify tumor mutations. Pre-surgery serum and plasma were taken from the patients in which mutation was found in the tumor tissue. Extracellular vesicles were isolated from the serum followed by the extraction of EV-DNA. CfDNA was extracted from the plasma. The mutations found in the tumor were used to detect the circulating tumor DNA using ultra-deep sequencing. We compared the sensitivity of mutation detection and allele frequency obtained in EV-DNA and cfDNA. Results: The sensitivity of mutation detection in EV-DNA and cfDNA was 61.90% and 66.67%, respectively. We obtained almost identical sensitivity of mutation detection in EV-DNA and cfDNA in each of the four stages of colon carcinoma. The total DNA concentration and number mutant copies were higher in cfDNA vs. EV-DNA (*p* value = 0.002 and 0.003, respectively). Conclusion: Both cfDNA and EV-DNA can serve as tumor biomarkers. The use of EV-DNA did not lead to improved sensitivity or better detection of tumor DNA in the circulation.

## 1. Introduction

Liquid biopsy has emerged as a promising diagnostic tool and consists of the identification of cancer cell content or free cancer cells outside the tumor mass. This content can be detected from blood, urine, stool, cerebrospinal fluid and other body fluids [1]. This minimally invasive procedure has a major advantage of being simple, rapid, easily available and less expensive with low risk for the patients. Compared to tumor biopsies, it is less expensive and more rapid, gives information on the complete molecular profile and heterogeneity of tumor and allows serial assessment to monitor the treatment response. 

Circulating tumor DNA (ctDNA) constitutes a small fraction of cell-free DNA (cfDNA) and can be distinguished from it by the presence of somatic mutation, copy number variations and epigenetic alterations that are characteristic of tumor cells [2]. In the last decade, ctDNA has emerged as one of the most important types of liquid biopsy and has been applied for cancer detection, follow up, disease relapse and for providing information on the molecular profile and heterogeneity of tumor [1]. One of the major challenges is the identification of the small fraction of ctDNA on the background of normal cell-free DNA. New ultra-sensitive detection techniques such as BEAMing (beads, emulsions, amplification and magnetics), droplet digital PCR and next-generation sequencing can help us overcome this hurdle [1].

Extracellular vesicles (EV) include a diverse population of membrane-bound vesicles released by various cells of our body. They carry cell-derived contents including proteins, lipids, metabolites, microRNA and nucleic acids and play a significant role in intercellular communication in both physiological and pathological conditions [3]. Based on their size and biogenesis, they have been categorized into subtypes including apoptotic bodies, microvesicles and exosomes [4]. The International Society for Extracellular Vesicles recommends designating all the different subtypes as EV since the available isolation methods do not lead to the complete separation of its subtypes and no specific biomarker is available for differentiating between them [5].

EV are relatively stable in the circulation or extracellular environment and found in several body fluids of cancer patients such as synovial fluid, cerebrospinal fluid, bronchial lavage fluid, breast milk, serum, saliva, urine, ascites and malignant effusions. Its diverse cargo, stability and ubiquitous presence has credited it as a promising biomarker in liquid biopsy. Proteins, microRNA and mRNA are the most investigated amongst its contents [6].

Recent studies have demonstrated that extracellular vesicles contain double-stranded DNA exhibiting the entire genome and comprising KRAS and TP53 mutants [7,8]. Moreover, the lipid bilayer protects the contents of EV from degradation; thus, EV-DNA are less fragmented and more stable under different storage conditions [8,9]. There is limited information regarding EV-DNA as a cancer biomarker and there is debate regarding its potential advantage over cfDNA. Klump et al. found that EV-DNA had a higher amount of DNA but lesser mutant alleles of BRAF and cKIT compared to cfDNA obtained from supernatant after EV isolation in advanced cases of melanoma patients [10]. A higher sensitivity of KRAS mutation detection was reported in EV-DNA than cfDNA in PDAC patients by Allenson and colleagues [11]. A similar study found that both cfDNA and EV-DNA had nearly the same KRAS mutation detection rate for localized and metastatic pancreatic cancer. However, concordance with the tissue sample was higher for EV-DNA than with cfDNA [12]. Wan et al. found EV-DNA to be superior compared to cfDNA in EGFR mutation detection in the early stage of non-small cell lung cancer. However, recent study on liquid biopsy of lung carcinoma patients found a higher mutant allele frequency of EGFR in cfDNA than EV-DNA [13]. Thus, it still remains under debate whether analysis of EV-DNA has any advantage over cfDNA in cancer detection.

In the present study, we used serum and plasma samples from patients with colon carcinoma to evaluate the sensitivity of EV-DNA and cfDNA for detecting tumor mutations in the circulation.

## 2. Materials and Methods

### 2.1. Patient Selection

Thirty-three cases of colon carcinoma were selected from amongst all the cases of colon carcinoma who had undergone surgical resections in Tel Aviv Sourasky Medical Center and had tumor tissue sample, serum and plasma samples available in the hospital’s biobank (Tel Aviv Biobank and MIDGAM-Israel National Biobank for Research). We excluded the cases in which either of three, i.e., tissue, plasma and serum was not available. This study was approved by national (Israel MOH) and Tel-Aviv Sourasky medical center local ethics committee (approval no: 0118-17-TLV, approval date: 31 January 2019).

### 2.2. Extraction of DNA from Tumor Tissue

Hematoxylin- and eosin-stained tissue sections of each sample were reviewed and the areas of tumor were marked. The fraction of tumor cells in the marked areas was estimated. Six sections of 8 µm thickness of formalin fixed paraffin embedded (FFPE) tissue samples were cut and areas corresponding to the representative marked areas were micro-dissected. DNA was extracted using the ReliaPrep™ FFPE gDNA Miniprep System (Promega, Madison, WI, USA) according to the manufacturer’s instructions. 

### 2.3. Panel Design

In order to maximize the possibility to find a key mutation in the tumor, we developed a genetic panel that covered the genomic regions with the highest mutation frequency in colon cancer in the “COSMIC” database [14]. For each position in the exome, we summed the mutations that were found in 45 bases before and after it and normalized them to the number of times each gene was sequenced in this database (on colon tumors). A PCR-based panel was designed to cover every position that had a 4% or above chance to have a mutation. The panel covers areas in the genes KRAS, TP53, APC, PIK3CA, BRAF, ACVR2A, RNF43 and TGFBR2 (Table S1). The panel was designed to work in a multiplex according to Biezuner et al. Genome Research 2016 [15], and the primers sequence were then tested using Thermo fisher “multiple primers analyzer” to reduce potential primer dimers. The primer pairs were split to three multiplexes mixes, primers that showed potential dimers or that amplify neighboring areas were assigned to different mixes. This panel showed uniformity of 96.88 ± 4.70% depending on the DNA quality; older samples tend to have less uniformity (Figure S1).

### 2.4. Isolation and Characterization of Extracellular Vesicles

EV were isolated from 500 to 1500 µL of pre-surgery serum samples of all the cases of colon carcinoma patients having mutation in the tissue DNA using Invitrogen Total Exosome Isolation (Vilnius, Lithuania) according to the manufacturer’s instructions. Hemolyzed serum samples were excluded. Size distribution analysis of EV extracted from two random samples was performed using dynamic light scattering with a ZetaSizer (Malvern Instruments, Worcestershire, UK). Samples were diluted in phosphate-buffered saline (PBS) and 3 × measurement runs were performed under standard settings (refractive index: 1.331, viscosity: 0.89, temperature: 25 °C). 

### 2.5. Extraction of EV-DNA and cfDNA from Plasma

DNA was extracted from EV isolated from serum and cfDNA was extracted from 900–2300 µL of pre-surgery plasma samples of all the cases of colon carcinoma showing mutation in tissue DNA using MagMAX™ Cell Free DNA Isolation Kit (Austin, TX, USA) according to the manufacturer’s instructions. Hemolyzed plasma samples were excluded. DNA was quantified using Qubit 3.0 fluorometer and Qubit™ ds DNA HS assay kit (Eugene, OR, USA) according to the manufacturer’s instructions.

### 2.6. Library Preparation for Next Generation Sequencing

An amplification-based sequencing method was used to construct libraries for sequencing using a two-step PCR protocol (Figure S2). In first step, the genomic segments were amplified using gene-specific primers. In each pair of primers, one was coupled to the M13 sequence and the other to the P1 sequence. In the second step, barcodes and adapters were attached to allow the binding to ion torrent sphere particles. To detect mutation in tumor tissue DNA, 19 genomic segments were amplified using gene-specific primer in three multiplex reactions (Table S1). The products of three multiplex reactions were consolidated and diluted with double-distilled water (DDW) to 1:100 dilution and 1 µL of it was used in the second PCR reaction. Each sample was tested in duplicate and each PCR was performed with its negative control to test the integrity of reagents. PCR products were cleaned with the kit: Wizard^®^ SV Gel and PCR Clean-Up System PROMEGA (Madison, WI, USA).

Amongst the mutations present in colon carcinoma cases, we chose those known to be driver mutations to be analyzed in the EV-DNA and cfDNA by using primers designed for specific mutation (Table S2). Each sample was tested for one mutation using the two-step PCR protocol. Each sample was tested in duplicate and along with its negative control. To determine the level of noise at a specific mutation position, we tested each primer with wild-type DNA. PCR products were cleaned with the AMPure XP PCR purification beads (Brea, CA, USA).

### 2.7. Sequencing Data Analysis

Sequencing was carried out using an Ion 510TM chip on the Ion Gene studio S5 for 500 flows (Thermo Fisher Scientific, Guilford, CT, USA). We aimed for at least 1000 coverage to allow the accurate determination of mutation fraction in each sample from tumor tissue DNA. The sequencing data from the machine were processed using the S5 Torrent server VM, which removed the adapter sequence, filtered poor quality reads and generated good-quality sequenced reads. We used variant caller software to determine the mutation in colon carcinoma cases. The variant caller files were downloaded, and mutations were characterized using the WANNOVAR website: http://wannovar.wglab.org (accessed date, 27 July 2021) [16]. Mutations were called if the allele frequency was > 5%, they had at least 10 reads and were present in both duplicates. 

For accurate determination of mutant fraction in EV-DNA and cfDNA, we aimed for at least 10,000 reads from each sample. The sequence files were aligned to the specific genomic sequence and the fraction of the mutation and the wild-type (WT) copies of the gene in each sample was determined using the Integrative Genomic Viewer (IGV2.4, http://software.broadinstitute.org/software/igv/; accessed on 27 July 2021) free software. Mutations were called when the mutant allele frequency (MAF) was at least three times higher than noise in wild-type and each duplicate had at least 10 reads coverage of mutant allele. Mutations were considered negative when they did not meet the criteria of positive mutation and had at least 5000 cumulative reads coverage from the duplicates. Results were regarded as inconclusive when criteria of positive mutation were not met and cumulative reads coverage from the duplicates were less than 5000. 

The number of mutant copies in cfDNA (mutants per mL of plasma) was calculated from mutant allele fraction and total DNA concentration in plasma. Similarly, the number of mutant copies in EV-DNA (mutants per mL of serum) was calculated from mutant allele fraction and total DNA concentration obtained from extra cellular vesicles derived from serum.

### 2.8. Statistical Analysis

Data analyses were carried out using Graph Pad Prism software. The statistical significance was determined by Fisher’s exact test, Mann–Whitney test and Wilcoxon matched pairs signed test. All tests conducted were two-sided and considered significant at *p* value * ≤0.05, ** ≤0.01, *** <0.001.

## 3. Results

Thirty-three cases of colon carcinoma were included in the study. The mean age at presentation was 69 ± 14.29 years and the male:female ratio was 1.7:2 (Table 1). The mean diameter of tumor was 4.59 ± 1.64 cm. The mean diameter of tumor was 4.59 ± 1.64 cm. A total of 10/33 (30.30%) cases included in the study were of stage I, 10/33 (30.30%) cases of stage II, 9/33 (27.27%) were of stage III and 4/33 (12.12%) were of stage IV and had metastasis.

**Figure 1:**
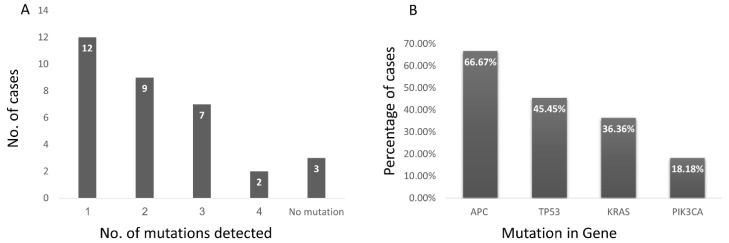
Mutation analysis in DNA extracted from FFPE tissue sections of colon carcinoma cases. (**A**) Bar chart shows number of mutations detected in cases of colon carcinoma using 19 amplicon gene panel. One mutation was detected in 12/33 cases, 2 in 9/33, 3 in 7/33, 4 in 2/33 and no mutation was detected in 3/33 cases of colon carcinoma. (**B**) Bar chart shows the prevalence of mutation in various genes in cases of colon carcinoma. The most common mutated gene was APC, present in 66.67% of cases followed by TP53 in 45.45%, KRAS in 36.36% and PIK3CA in 18.18%.

In the tissue samples, coverage analysis of gene panel showed uniformity of 96.88 ± 4.70% and 99th percentile of samples in duplicates had 99.53 reads. One to four mutations were detected in 30 out of 33 (90.90%) cases (Figure 1A). APC was the most commonly mutated gene, present in 22 (66.67%) followed by TP53 in 15 (45.45%), KRAS in 12 (36.36%) and PIK3CA in 6 (18.18%) (Figure 1B).

### 3.1. Extra Cellular Vesicles Characterization

Dynamic light scattering was used to measure the size distribution of EV and demonstrated that the average size of EV isolated from two samples was 82.8 ± 14.6 nm and 101.0 ± 1.4 nm (Figure S3)

### 3.2. Blood cfDNA Concentration Is Higher Than EV-DNA

**Figure 2:**
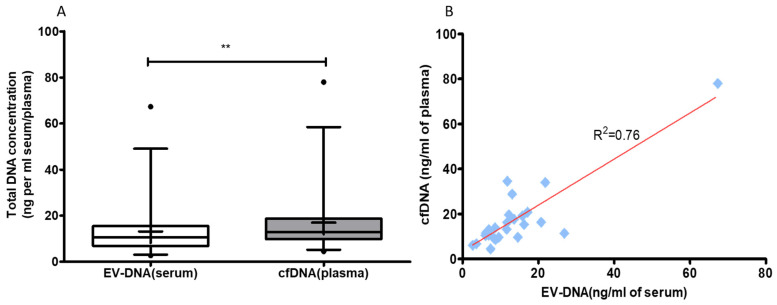
Total concentration of EV-DNA and cfDNA from colon carcinoma cases. (**A**) Box plot shows the comparison of total DNA concentration in EV-DNA and cfDNA. The total DNA concentration was more in cfDNA than EV-DNA. (*p* value = 0.002). Wilcoxon matched pairs signed test; whiskers represents 5th and 95th percentile and mean shown by + in box plot. (**B**) Scatter plot shows correlation between total concentration of EV-DNA and cfDNA (*p* value < 0.001, R^2^ = coefficient of determination; blue squares represent EV-DNA and cfDNA concentration). ** refers to *p* ≤ 0.01.

EV-DNA and cfDNA were extracted from EV obtained from the serum and plasma, respectively, of 28 cases of colon carcinoma. Two cases with hemolyzed serum and plasma samples were excluded from the study. The total concentration of EV-DNA ranged from 2.69 to 67.32 ng/mL (median = 10.59 and mean ± SD = 13.19 ± 12.03 ng/mL of serum). The total concentration of cfDNA obtained from plasma ranged from 4.49 to 77.98 ng/mL of plasma (median = 12.89 and mean ± SD = 16.97 ± 14.10 ng/mL of plasma). The cfDNA obtained from plasma was significantly higher than EV-DNA (*p* value = 0.002; Figure 2A). We found a significant correlation between the concentrations of EV-DNA and cfDNA (R^2^ = 0.76, *p* value < 0.001; Figure 2B). The mean and median concentration of both EV-DNA and cfDNA was higher in late-stage (III and IV) compared to that in early-stage (I and II) disease. The difference was statistically significant in EV-DNA (median = 7.99 ng/mL versus 17.16 ng/mL; *p* value = 0.02, Mann–Whitney test) but not in cfDNA (median = 12.89 ng/mL versus 13.85 ng/mL; *p* value = 0.94, Mann–Whitney test). No correlation between the size of tumors and concentration of DNA in both EV and plasma was noted (R^2^ = 0.013).

### 3.3. The Sensitivity of Tumor DNA Detection Was Similar in EV-DNA and cfDNA

**Figure 3:**
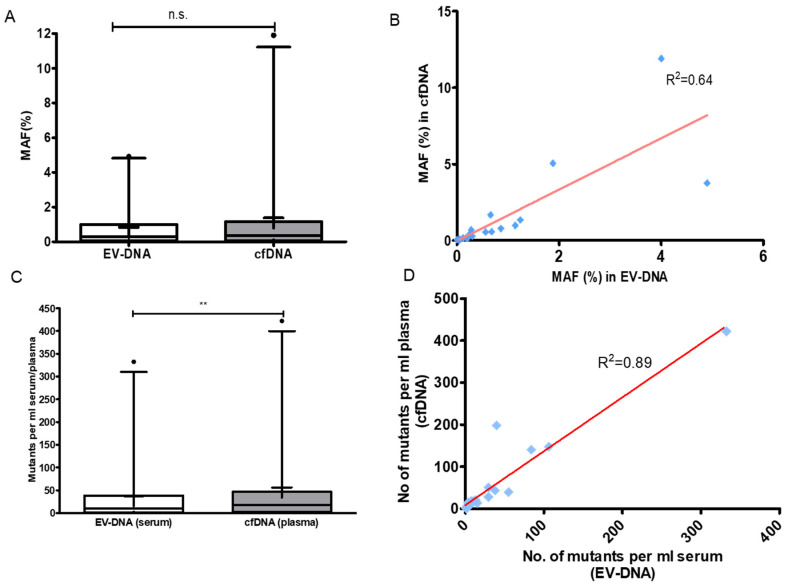
Comparison of mutant allele frequency (MAF) and mutant per mL serum/plasma in EV-DNA vs. cfDNA. (**A**) Box plot to show the comparison of MAF in EV-DNA and cfDNA. No significant difference was found between them (*p* value = 0.316; Wilcoxon matched pairs signed test, n.s.—not significant). Whiskers represent 5th and 95th percentile and mean shown by + in box plot. (**B**) Scatter plot shows correlation between MAF in EV-DNA and cfDNA (*p* value < 0.001, R^2^ = coefficient of determination, blue squares represent MAF in EV-DNA and cfDNA). (**C**) Box plot shows that the number of copies of mutants were more in cfDNA than in EV-DNA (*p* value = 0.003; Wilcoxon matched pairs signed test, Whiskers represent 5th and 95th percentile and mean shown by + in box plot). (**D**) Scatter plot shows correlation between number of copies of mutants in cfDNA and EV-DNA *(p* value < 0.001, R^2^ = coefficient of determination, blue squares represent mutants per mL serum/plasma). ** refers to *p* ≤ 0.01.

**Figure 4:**
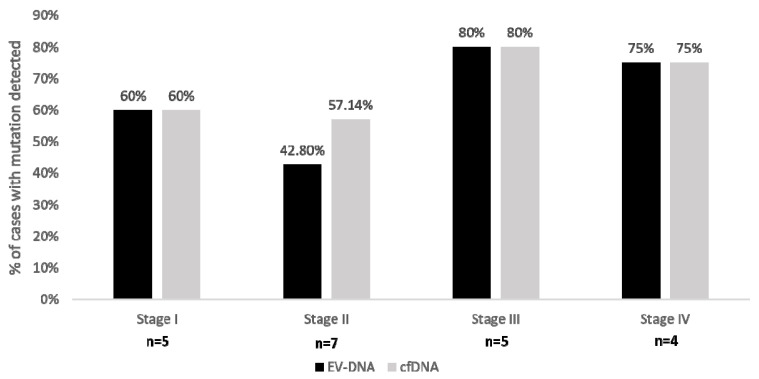
Comparison of mutation detection in EV-DNA and cfDNA in the four stages of colon carcinoma. Bar chart shows that the mutation detection was almost equivalent in EV-DNA and cfDNA for all the four stages of colon carcinoma (*p* value = 1; Fisher’s exact test).

Mutation analysis was performed in EV-DNA and cfDNA obtained from 27 cases. In one of the cases, we detected frameshift mutation in RNF43 at position G659Vfs*40. It had seven consecutive cytosine nucleotides at the mutation site. Since it is difficult to detect mutations involving more than the same six consecutive bases in the ion torrent platform [17], this case was dropped out and mutation analysis in EV-DNA and cfDNA was performed in 27 out of 28 cases. Two out of twenty-seven had a germline variant in the APC gene and were excluded from further analysis. The average mutant allele frequency (MAF) obtained in EV-DNA and cfDNA was 0.83 ± 0.01% and 1.37 ± 0.03% with a median of 0.28% and 0.37%, respectively. There was no significant difference between MAF in EV-DNA and cfDNA (*p* value = 0.316; Figure 3A). A high correlation was found between MAF in EV-DNA and cfDNA (R^2^ = 0.64, *p* value < 0.001; Figure 3B). The number of mutant DNA fragments were higher in cfDNA compared to EV-DNA (median = 17.92 mutants per mL of plasma vs. 10.60 mutants per mL of serum, *p* value = 0.003; Figure 3C). There was a significant correlation between the number of copies of mutants in cfDNA and EV-DNA (R^2^ = 0.89, *p* value < 0.001; Figure 3D). Four out of 25 samples gave inconclusive results in both EV-DNA and cfDNA as we could not achieve 5000 cumulative reads from the duplicates. Tumor mutations were detected in 13/21 (61.90%) cases in EV-DNA and in 14/21 (66.67%) cases in cfDNA (Table 2). We found a trend towards increased mutation detection in late-stage (III and IV) compared to early-stage (I and II) disease: 7/9(77.77%) in both EV-DNA and cfDNA versus 6/12 (50%) in EV-DNA and 7/12 (58.33%) in cfDNA, (*p* value = 0.37 and 0.64, Fisher’s exact test). Mutation detection rate was almost identical in EV-DNA and cfDNA in all the four stages: 3/5 (60%) in stage I, 4/5 (80%) in stage III, 3/4 (75%) in stage IV in both EV-DNA and cfDNA; 3/7 (42.80%) and 4/7 (57.14%), respectively, in EV-DNA and cfDNA in stage II (Figure 4). The frequency of mutation detection was higher in large size tumors. Mutation was identified in 1/6 (16/66%) in both EV-DNA and cfDNA when tumor diameter was <4 cm; and in 12/15 (80%) and 13/15 (86.67%) in EV-DNA and cfDNA, respectively, when tumor diameter was ≥4 cm (Table 2, *p* value = 0.0139 and 0.0055; Fisher’s exact test).

## 4. Discussion

CfDNA consists of fragments of DNA released into the circulation mostly by apoptotic and necrotic cells [18]. It is highly fragmented double-stranded DNA of varying size ranging from 120 to 220 base pairs and a peak of 167 base pairs, suggesting apoptosis to be the main mechanism of its release [19]. Its presence was first reported in 1948 [20]; however, it took years of extensive research, coupled with advances in molecular techniques, to establish its utility in liquid biopsy [21]. Numerous studies have demonstrated its potential role as a cancer biomarker for diagnosis and follow-up of disease, as well as in the detection of treatment resistance and to monitor the treatment response [22]. The recent discovery of the presence of double-stranded DNA associated with EV has raised an open question of whether it has any advantage over cfDNA as a cancer biomarker. The main objective of liquid biopsy is to obtain a sensitive, efficient, reliable and cost-effective method which would help in early diagnosis of cancer. Therefore, additional investment in terms of cost, time and labor on EV isolation, followed by DNA extraction, is worth incurring if it provides an added value and has better sensitivity in cancer detection compared to cfDNA.

In this study, we identified mutation in 50% and 58.33% of cases, respectively, in EV-DNA and cfDNA in early-stage (I and II) disease and 77.77% of cases in both EV-DNA and cfDNA in late-stage (III and IV) disease. The sensitivity of mutation detection was similar to previously reported studies [2,23]. There was a trend towards increased mutation detection in late-stage (III and IV) disease, compared to early-stage (I and II) disease, in both EV-DNA and cfDNA. However, the difference was not statistically significant, probably due to the small cohort size, as well as because only four cases with metastasis were included in the study. A higher frequency of mutation detection had been earlier reported in large-sized and advanced tumors [23,24]. We also found a significant increase in the frequency of mutation detection when the diameter of tumor was ≥4 cm in both EV-DNA and cfDNA.

We found equivalent sensitivity of mutation detection in both EV-DNA and cfDNA (61.90% and 66.67%) obtained from patients of colon cancer. Our findings were in agreement with previous studies which had also reported similar sensitivity of mutation detection in both EV-DNA and cfDNA [12,25]. We obtained almost identical sensitivity of mutation detection in EV-DNA and cfDNA in each of the four stages of colon carcinoma (Figure 4). Similarly, Bernard et al. reported nearly equivalent sensitivity of mutation detection in both cfDNA and EV-DNA for localized and metastatic pancreatic cancer [12]. However, Allenson and colleagues noted a higher sensitivity in EV-DNA compared to cfDNA in localized, locally advanced, and metastatic pancreatic adenocarcinoma patients [11]. Wan et al. found EV-DNA to be superior to cfDNA in the detection of early-stage non-small cell lung cancer; however, no advantage was noted in late-stage tumors [26]. The difference in the results could be attributed to various technical issues, such as a lack of standardized procedure for EV isolation, different methods employed for DNA extraction and different techniques utilized for mutation detection.

In concordance with previous studies, we found that the total concentration of cfDNA obtained from plasma was significantly higher than EV-DNA [26,27]. While there was no significant difference between MAF obtained in EV-DNA and cfDNA, the number of copies of mutants were more in cfDNA. There was a high correlation between the total DNA concentration, MAF and number of copies of mutants in EV-DNA and cfDNA. These findings were in line with earlier studies suggesting that a proportion of cfDNA was associated with different subtypes of EV [27,28]. However, the subtype contributing the most is not yet known and is under investigation [29]. A higher number of copies of mutants in cfDNA can be ascribed to the higher concentration of DNA obtained from plasma. Few earlier studies have reported a higher number of copies of mutant allele in cfDNA obtained from the supernatant left after extraction of EV than EV-DNA [10,13].

One of the obstacles in the assessment of cfDNA is the decreased ability of NGS- and PCR-based approaches to cover all its small fragments of varying sizes. This results in PCR amplification failure and difficulties in library preparation, leading to less sequenced reads [30,31]. EV-DNA is less fragmented due to the protection offered by the lipid bilayer from degradation; hence, this was perceived to be helpful in overcoming the above-mentioned problem. However, four of our samples could not give conclusive results due to low number of sequenced reads obtained from both cfDNA and EV-DNA. The analysis of EV-DNA did not provide the advantage of acquiring more sequenced reads than cfDNA. This suggests that the extraction of cfDNA from plasma also acquires DNA associated with EV. Thus, cfDNA is more efficient and cost-effective than EV-DNA in cancer detection, as the procurement of EV-DNA not only requires more steps, time and labor, but also loses the fraction of DNA and mutants present in blood in free-circulating form.

To conclude, our study showed that both EV-DNA and cfDNA had similar sensitivity of mutation detection in all of the four stages of colon carcinoma. Since the total DNA concentration and number of copies of mutants were significantly higher in cfDNA than EV-DNA, cfDNA can be considered a more efficient biomarker for the detection of colon cancer.

## Figures and Tables

**Table 1:** Patient characteristics of colon carcinoma patients.

**Age (Years)**	Mean ± SD		69 ± 14.29
**Sex**	Male		15 (45.45%)
	Female		18 (54.54%)
**Diameter (cm)**	Mean ± SD		4.59 ± 1.65
**Grade (Differentiation)**	Well		7 (21.21%)
	Moderate		20 (60.60%)
	Poor		3 (9.09%)
	Mucinous		3 (9.09%)
**Stage**	T	T1	1 (3.03%)
		T2	11 (33.33%)
		T3	18 (54.54%)
		T4	3 (9.09%)
	N	N0	22 (66.66%)
		N1	4 (12.12%)
		N2	7 (21.12%)
	I		10 (30.30%)
	II		10 (30.30%)
	III		9 (27.27%)
	IV		4 (12.12%)
**Metastasis**	Yes		4 (12.12%)
	No		29 (87.87%)
**Vascular Invasion**	Yes		7 (21.12%)
	No		16 (48.48%)

**Table 2:** Mutation analysis from EV-DNA and cfDNA.

Serial No	Gene	Mutation	Stage	Tumor Size (cm)	MAF in EV-DNA (%)	No. of Mutant Reads from Each Duplicate	MAF in cfDNA (Plasma) (%)	No. of Mutant Reads from Each Duplicate	Noise in Wild Type DNA (%)
1	*TP53*	p.R175H (c.524G>A)	II	2.5	0.30%	≥10	0.29%	≥10	0%
2	*KRAS*	p.G12V (c.35G>T)	II	2.5	0.05%	<10	0.04%	<10	0.008%
3	*APC*	p.R1450X (c.4348C>T)	III	4	1.14%	≥10	0.99%	≥10	0%
4	*TP53*	p.R248W (c.742C>T)	IV	4.5	4.00%	≥10	11.90%	≥10	0%
5	*TP53*	p.R273H (c.818G>A)	IV	6	1.24%	≥10	1.34%	≥10	0.026%
6	*KRAS*	p.G12S (c.34G>A)	IV	5.5	0.24%	≥10	0.37%	≥10	0.012%
7	*APC*	p.E1309X (c.3925G>T)	IV	2.5	0.04%	<10	0.02%	<10	0.00%
8	*PIK3CA*	p.E545K (c.1633G>A)	II	6	0.68%	≥10	0.57%	≥10	0%
9	*PIK3CA*	p.H1047L (c.3140A>T)	II	6	4.90%	10	3.75%	≥10	0%
10	*TP53*	p.C242R (c.724T>C)	I	6	NA	NA	NA	<10	0.05%
11	*KRAS*	p.G12D (c.35G>A)	II	2.5	0%	<10	0.05%	<10	0.014%
12	*KRAS*	p.G12V (c.35G>T)	II	2	0.05%	<10	0.07%	<10	0.008%
13	*TP53*	p.R175H (c.524G>A)	III	8	0.66%	≥10	1.68%	≥10	0%
14	*APC*	p.Q1406X (c.4216C>T)	III	5.2	0.86%	≥10	0.78%	≥10	0.09%
15	*TP53*	p.G245S (c.733G>A)	I	5	0.56%	≥10	0.56%	≥10	0.02%
16	*TP53*	p.A161T (c.481G>A)	I	1.2	0.06%	<10	0.06%	<10	0%
17	*TP53*	p.M237V (c.709A>G)	I	6	0.04%	<10	0.06%	<10	0%
18	*TP53*	p.R175H (c.524G>A)	III	5.5	0.18%	<10	0.18%	<10	0%
19	*TP53*	p.R306X (c.916C>T)	I	1	NA	NA	NA	NA	0.06%
20	*APC*	p.R232X (c.694C>T)	III	6	0.28%	≥10	0.69%	≥10	0.07%
21	*APC*	p. P1432Hfs*4	I	4.5	1.88%	≥10	5.05%	≥10	0.24%
22	*TP53*	p.R175H (c.524G>A)	I	3	NA	NA	NA	NA	0%
23	*APC*	p.R1450X (c.4348C>T)	II	4	0.12%	<10	0.17%	≥10	0%
24	*TP53*	p.R175H (c.524G>A)	II	6	NA	NA	NA	NA	0%
25	*KRAS*	p.G12V (c.35G>T)	I	5	0.21%	≥10	0.17%	≥10	0.008%

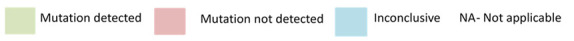

## Data Availability

Data available on request due to privacy restrictions.

## Supplementary Materials

The following are available online at https://www.mdpi.com/article/10.3390/genes12081171/s1, Figure S1: Coverage analysis of gene panel. Figure S2: The Two Step PCR protocol for library preparation. Figure S3 Size distribution analysis of EV. Table S1: Specific primers for 19 genomic regions of hotspot panel. Table S2: Gene specific primer for liquid biopsy.
