# Supplementary material for: Extracellular Vesicle-Derived DNA vs. CfDNA as a Biomarker for the Detection of Colon Cancer
Source: Genes (Basel). 2021 Jul 29;12(8):1171. doi: 10.3390/genes12081171 (PMC8393490; doi:10.3390/genes12081171)
Supplement: Supplementary file 1 [file genes-12-01171-s001.zip › genes-1287916-supplementary.pdf]

Supplementary Materials:

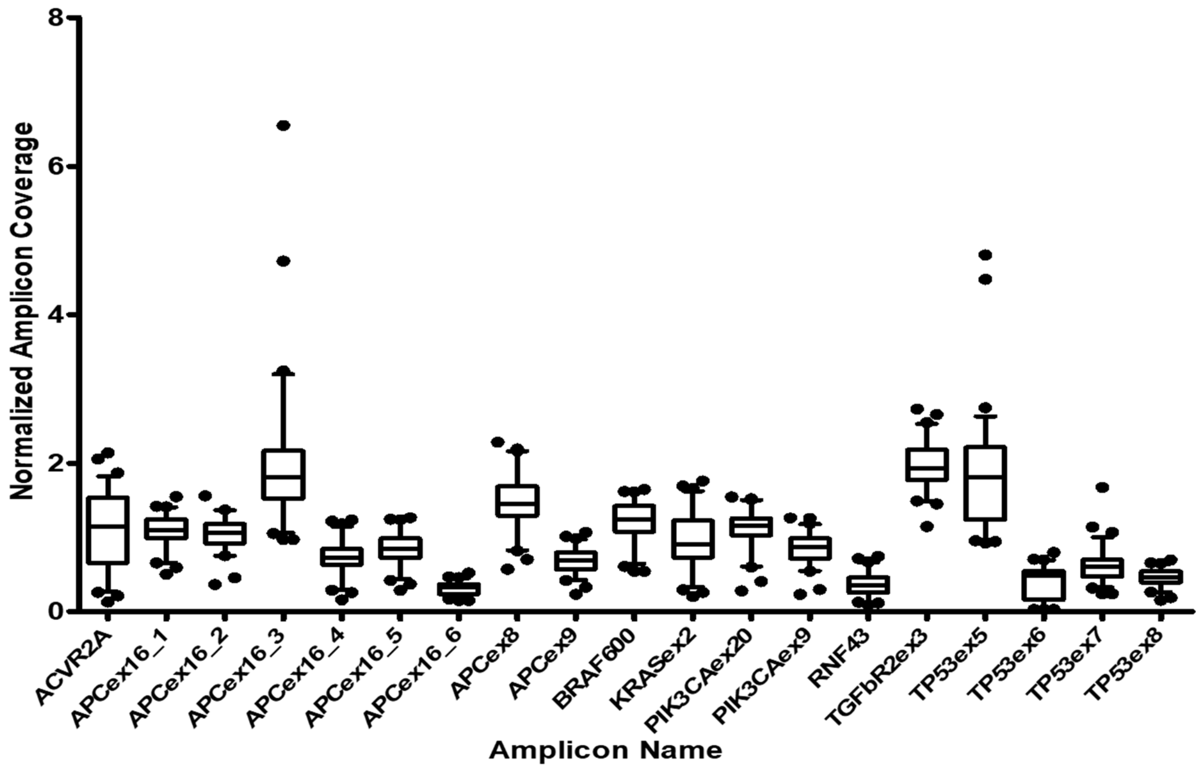

Figure S1. Coverage analysis of gene panel. Box plots shows normalized amplicon coverage of 19 amplicon gene panel (Whiskers represents 5th and 95th percentile). The panel had a uniformity of 96.88±4.70%.

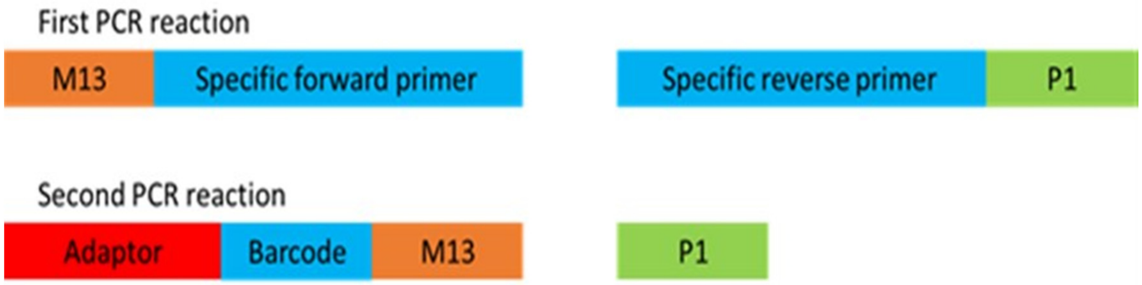

Figure S2. The Two Step PCR protocol for library preparation.

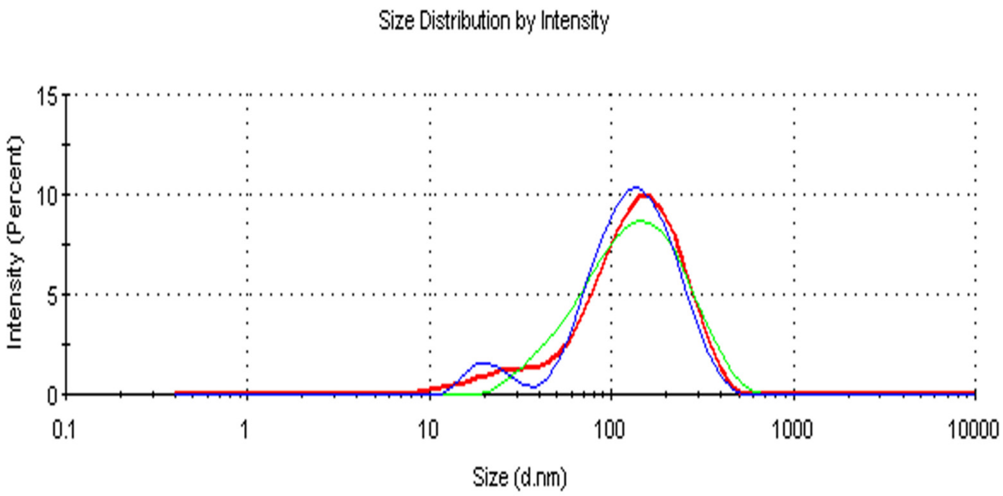

Figure S3. Size distribution analysis of EV. Graph shows results of 3X measurement runs of a sample by diffuse light scattering; the average size of EV was 101.0±1.4 nm.

Table S1. Specific primers for 19 genomic regions of hotspot panel.

Primer mixture for multiplex	Gene	Chromosome: Gene coordinate[a]	Forward Primer (Name) Sequence	Reverse Primer (Name) Sequence	Amplicon size
1	*ACVR2A*	chr2:148683660+148683815	(HS_ACVR2A_F) AAGACATGCAGGAAGTTGTT	(HS_ACVR2A_R) AGAAAGAGAAATGTGCAGAGATA	156
	APC	chr5:112175116+112175320	(HS_APC_ex16_1_F) TTCATTATCATCTTTGTCATCAG	(HS_APC_ex16_1_R) AGATAAACTAGAACCCTGCAGTC	205
		chr5:112175398+112175606	(HS_APC_ex16_3_F) CAAAAGTCCACCTGAACACTAT	(HS_APC_ex16_3_R) GTGTTTTACTTCTGCTTGGTG	209
		chr5:112175540+112175757	(HS_APC_ex16_6_F) ATTATAAGCCCCAGTGATCTT	(HS_APC_ex16_6_R) AATAAAGTATCAGCATCTGGAAG	218
		chr5:112128030+112128245	(HS_APC_ex9_F) AATGAGAATGATTTGACATAACC	(HS_APC_ex9_R) AGAAAGGCAATTTACTAACCTCT	216
	PIK3CA	chr3:178935914+178936125	(HS_PIK3CA_ex9_F) TTTGCTTTTTCTGTAAATCATCT	(HS_PIK3CA_ex9_R) TACCTGTGACTCCATAGAAAATC	212
	TP53	chr17:7576970-7577179	(HS_TP53_ex8_F) TTGCTTCTCTTTTCCTATCCT	(HS_TP53_ex8_R) ATAACTGCACCCTTGGTCTC	210
2	*APC*	chr5:112116474+112116644	(HS_APC_ex8_F) TTTGTGGTTTTAGTTTTCCTTAC	(HS_APC_ex8_R) TAATAGCTCTTCGCTGTTTTATC	171
		chr5:112175665+112175875	(HS_APC_ex16_4_F) TACTGCTGAAAAGAGAGAGAGTG	(HS_APC_ex16_4_R) AACTGGAGGCATTATTCTTA	211
		chr5:112175267+112175472	(HS_APC_ex16_5_F) GTGTCACAGCACCCTAGAAC	(HS_APC_ex16_5_R) TCAAGTGAACTGACAGAAGTACA	205
		chr5:112175853+112176066	(HS_APC_ex16_2_F) AATTAAGAATAATGCCTCCAGTT	(HS_APC_ex16_2_R) TTTGCTTTACGTGATGACTTT	214
	TP53	chr17:7578286-7578500	(HS_TP53_ex5_F) CAGCTGTGGGTTGATTCC	(HS_TP53_ex5_R) AGACCTAAGAGCAATCAGTGAG	215
		chr17:7578215-7578382	(HS_TP53_ex6_F) CAGATAGCGATGGTGAGC	(HS_TP53_ex6_R) AAGTGTTTCTGTCATCCAAATAC	168

	chr17:7577434-7577633	(HS_TP53_ex7_F) ATCTTGGGCCTGTGTTATC	(HS_TP53_ex7_R) GGTCAGAGGCAAGCAGAG	200
3	*APC*	chr5:112175853+112176066	(HS_APC_ex16_2_F) AATTAAGAATAATGCCTCCAGTT	(HS_APC_ex16_2_R) TTTGCTTTACGTGATGACTTT	214
	BRAF600	chr7:140453055-140453265	(HS_BRAF600_F) CTAAACTCTTCATAATGCTTGCT	(HS_BRAF600_R) AAAATAGCCTCAATTCTTACCAT	211
	KRAS	chr12:25398151-25398344	(HS_KRAS_ex2_F) TATTTTTATTATAAGGCCTGCTG	(HS_KRAS_ex2_R) TATCTGTATCAAAGAATGGTCCT	194
	PIK3CA	chr3:178951941+178952138	(HS_PIK3CA_ex20_F) TAAATCTTTTCTCAATGATGCTT	(HS_PIK3CA_ex20_R) GCTGTTTAATTGTGTGGAAGAT	198
	RNF43	chr17:56434996-56435211	HS_RNF43_F GCAAAAATCCAGCCTCTCTG	HS_RNF43_R GTTTCTGGTAGCAGCCTCTT	216
	TGFBR2	chr3:30691821+30691975	(HS_TGFBR2_ex3_F) ACCATGACTTTATTCTGGAAGAT	(HS_TGFBR2_ex3_R) ACCCTTAAGAGAAGAAAACTCAC	155

a – Gene coordinates correspond to Human genome version hg19.

Table S2. Gene specific primer for liquid biopsy.

Gene	Chromosome: Gene coordinate[a]	Forward Primer (Name) Sequence	Reverse Primer (Name) Sequence	Amplicon size
APC	chr5:112175149+112175239	(APC1307_F) AATAGGATGTAATCAGACGACAC	(APC1307_R) AGCTGACCTAGTTCCAATCTT	91
	chr5:112175468+112175546	(APC1406_F) CTTGATAGTTTTGAGAGTCGTTC	(APC1406_R) TTATAATGCCACTTACCATTCC	79
	chr5:112128164+112128245	(APC_232F) CAAATCGAAAAGGACATACTTC	(APC_232R) AGAAAGGCAATTTACTAACCTCT	82
	chr5:112175540+112175610	(APC_1432F) ATTATAAGCCCCAGTGATCTT	(APC_1432R) GGAGGTGTTTTACTTCTGCTT	71
KRAS	chr12:25398240-25398313	(m13_KRASs_F) TGAATATAAACTTGTGGTAGTTG	(P1_KRAS_LB_R) GATTCTGAATTAGCTGTATCGTC	74
PIK3CA	chr3:178936057+178936126	(m13_lbPIK3ca545_F) AATTTCTACACGAGATCCTCTCT	(p1_lbPIK3ca545_R) TTACCTGTGACTCCATAGAAAAT	70
	chr3:178952021+178952138	(m13_lbPIK3ca1047_F) CTAGCCTTAGATAAAACTGAGCA	(p1_lbPIK3ca1047_R) GCTGTTTAATTGTGTGGAAGAT	118
TP53	chr17:7578426+7578500	(TP53_158_F) GTGCTGTGACTGCTTGTAGAT	(TP53_158_R) CAGCTGTGGGTTGATTCC	75
	chr17:7576996+7577079	(TP53_ex8end_F) CGCTTCTTGTCCTGCTTG	(TP53_ex8end_R) GAGAATCTCCGCAAGAAAG	84
	chr17:7578363-7578449	(M13TP53_R175H_F) GCCATCTACAAGCAGTCACA	(P1TP53_R175H_R) CTGCTCACCATCGCTATCTG	87
	chr17:7577491-7577580	(m13TP53_242F) ACAACTACATGTGTAACAGTTCT	(p1TP53_257R) CTCCTGACCTGGAGTCTTC	90
	chr17:7577071-7577164	(m13TP53_266t282F) CCTGAGTAGTGGTAATCTACTGG	(p1TP53_266to282R) GAGATTCTCTTCCTCTGT	94
	chr17:7577499+7577595	(TP53_237_F) CTGGAGTCTTCCAGTGTGAT	(TP53_237_R) GTACCACCATCCACTACAACT	97

a – Gene coordinates correspond to Human genome version hg19.
